# Cell Membrane-Camouflaged Nanoparticles: A Promising Biomimetic Strategy for Cancer Theragnostics

**DOI:** 10.3390/polym10090983

**Published:** 2018-09-03

**Authors:** Veena Vijayan, Saji Uthaman, In-Kyu Park

**Affiliations:** 1 Department of Biomedical Sciences, Chonnam National University Medical School, 160 Baekseo-ro, Gwangju 61469, Korea; veenavj4392@gmail.com; 2 Department of Polymer Science and Engineering, Chungnam National University, 99 Daehak-Ro, Yuseong-Gu, Daejeon 34134, Korea; sajiuthaman@gmail.com

**Keywords:** biomimetic, membrane-coated nanoparticle, immune escape, tumor therapy, homotypic targeting

## Abstract

Biomimetic functionalization of nanoparticles through camouflaging with cellular membranes has emerged as a promising strategy for cancer theragnostics. Cellular membranes used for camouflaging nanoparticles are generally isolated from blood cells, immune cells, cancer cells, and stem cells. The camouflaging strategy of wrapping nanoparticles with cellular membranes allows for superior tumor targeting through self-recognition, homotypic targeting and prolonged systematic circulation, thereby aiding in effective tumor therapy. In this review, we emphasized the various types of cellular membrane-camouflaged nanoparticles, their mechanisms in targeted therapy and various biomimetic strategies for anti-cancer therapy.

## 1. Introduction

Cancer has become one of the world’s most devastating diseases, with an estimated 1.7 million cases in 2018 [1]. Over the past few decades, progress towards cancer treatment modalities has greatly advanced and includes chemotherapy [2,3], radiotherapy [4], and surgical removal of tumor tissues [5]. These treatment modalities remain unsuccessful to a certain extent, as they cause undesired damage to healthy tissue and have low targeting ability towards cancer, resulting in poor therapeutic outcomes [6]. To solve this issue, an ideal tumor-specific delivery system needs to be designed to provide prolonged circulation in the body with specific targeting towards tumors. Targeted delivery could be achieved by active or passive targeting approaches. In passive targeting, the therapeutic agent is incorporated into a nanoparticle/macromolecule that passively reaches the target organ due to an enhanced permeation and retention (EPR) effect and nanosystem charge [7,8]. In the case of active targeting, the therapeutic agent or the carrier system is conjugated to tissue or cell-specific ligands, which are selected to bind to specific receptors overexpressed on tumors; for example, hyaluronic-based nanoparticles targeting to tumors via CD 44 ligand binding [9]. Active targeting occurs only when the therapeutic cargo nears the target to take advantage of its increased affinity and avidity [10]. Active targeting becomes difficult in the case of the delivery of membrane-impermeable drugs for targeting blood-borne diseases, which could be solved with the use of biomimetic nanoparticles. Biomimetic nanoparticles (NPs) mimic biological membranes and are increasingly used to achieve prolonged circulation, evasion of immune responses, and homologous targeting to tumor cells after administration in the body. Liposomes are biomimetic products that are generally used to mimic biological membranes. They are made by dispersing phospholipids in water and are known for higher loading capability and co-delivery of both hydrophilic and hydrophobic drugs [11]. Another biomimetic approach is coating the nanoparticles with cell membranes in order to provide nanoparticles with cell-like behaviors. This approach possesses several advantages, such as prolonged circulation [12,13,14], immune escape [15,16,17] and increased targeting abilities [18,19,20]. Membrane coating acts as a bridge to functionalize synthetic nanoparticles and makes it a suitable delivery vehicle for various biomedical applications [18,21,22,23]. The cell membrane-camouflaged nanoparticles will have a “core–shell structure” in which the nanoparticle (core) would be coated by a membrane (shell), derived from source cells through a series of ultracentrifugation and extrusion techniques, that has the same innate properties of “self- recognition” as their source cells. The cell membranes offer a double layer medium (due to the lipid bilayer structure) that allows transmembrane protein attachment with no loss in the functionalities and reliability during drug formulation for drug delivery. All biologically relevant moieties, such as membrane-bound antigens needed for immune evasion and targeting, are translocated onto the membrane coated over the nanoparticle. Different source cell membranes are used for coating nanoparticles, which makes them suitable for diverse applications in the field of tumor theragnostics [24,25,26,27]. The preferential accumulation of membrane-coated nanoparticles in the tumor site improves the efficacy of antitumor therapy as well as reducing the systemic toxicity [28,29]. The first reported membrane-coated nanoparticles (NPs) were of red blood cell (RBC) membranes coated onto negatively charged polymeric nanoparticles through extrusion [30]. To date, many types of membranes have been used for constructing cell membrane-camouflaged nanoparticles, including RBCs [12,13,14], leukocytes [31,32], neutrophils [33], platelets [34], macrophages [25], cytotoxic T cells [35], stem cells [36], and cancer cells [15,19,37]. This review summarizes the different types of cell membrane-camouflaged nanoparticles, their mechanism of camouflaging and applications in the field of cancer theragnostics.

## 2. Components of Cell Membrane-Camouflaged Nanoparticles (NPs)

**Figure 1:**
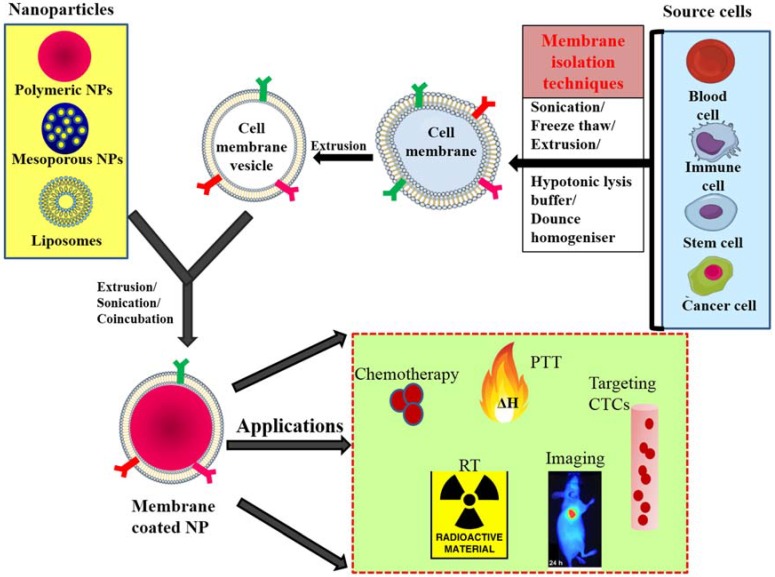
General scheme of preparation of membrane-coated nanoparticle and its biomedical applications; membranes isolated from different source cells by various methods and coating this onto core NPs by coincubation, sonication or extrusion. PTT—photothermal therapy, CTC—circulating tumor cells, RT—radiotherapy, NP—nanoparticle.

Cell membrane-camouflaged NPs normally comprise a therapeutic nanoparticle coated by a thin layer of cellular plasma membrane, thus forming a “core–shell” structure in which the nanoparticle is the core and the cell membrane is the shell. The core nanoparticle carries the payload that needs to be delivered to the desired site. Membranes obtained from different source cells are isolated through a series of ultracentrifugation techniques and coated onto nanoparticles via extrusion, sonication and electroporation techniques. After coating over the nanoparticle, the membrane proteins that were present on the membranes of source cells are translocated onto the surface of the newly coated nanoparticle and provide immune evasion abilities, prolonged circulation, and tumor targeting (Figure 1) [28,29,38].

### 2.1. Source Cells Used for Coating

Different types of source cells used for camouflaging are RBCs [12,13,39], white blood cell (WBCs) [31], platelets [34,40] macrophages [25,41], Natural Killer (NK) cells [26], cytotoxic T lymphocytes (CTLs) [35], neutrophils [33], stem cells [36] and cancer cells [37,42]. RBCs are the most important blood cells and are involved in supplying oxygen to the body. RBCs have a long circulation life of approximately 100–120 days before being cleared from the body via the immune system. Therefore, nanoparticles coated with RBC membranes provide prolonged circulation, thus preventing early uptake by the reticuloendothelial system (RES). RBCs have “self-markers” on their surfaces that prevent immune attack and make them suitable for use as long-circulating carriers. Another type of blood cell is platelets, which have innate properties of binding injured blood vasculature, immune-compatibility and adhesion to pathogens [43]. Platelet membrane-camouflaged nanoparticles enable the nanoparticles to escape immune system attack. The platelet membrane-coated nanoparticles bind to damaged blood vessels and certain pathogens, allowing the core nanocarrier to deliver the payload. Platelets contain unique surface moieties that facilitate subendothelial adhesion, pathogen interactions and immune evasion [20,34,40]. Another type of blood cell is the leukocyte, which is a type of WBC that forms a part of the immune system and is involved in protecting the body against contagious diseases and foreign invader [32]. Hence, leukocyte membranes have the property of immune system evasion and target tissue localization, thus exhibiting their targeting ability via cell–cell interactions [31,32]. Nanoparticles camouflaged with leukocyte membranes escape opsonization and delayed uptake via the mononuclear phagocyte system [31]. Macrophages are immune cells that identify, engulf and digest cellular debris and other foreign substances that lack the self-biomarkers of healthy cells. Macrophages are present in the tumor microenvironment by direct association with tumor progression and metastasis [44]. Nanoparticles camouflaged with macrophage membranes facilitate cell–cell adhesion for cancer targeting. Another type of immune cell is the neutrophil, which are granulocytes that are abundantly present in white blood cells. Neutrophils are not confined to specific areas of circulation and move freely through walls of veins and body tissues to instantly attack antigens. Neutrophils play a crucial role in tumor progression. They are recruited to tumors through secretion of chemoattractant from tumors. Another type of immune cell is the NK cells, which are large, granular lymphocytes that provide host defense against infections and tumor cells with abnormal expression of Major Histocompatibility Complex Class 1 (MHC-1) molecules and markers for cellular stress [45]. NK cells target cancer cells directly through inhibitory and activating receptors on their surfaces and can also induce cell killing without prior sensitization. Another type of immune cell, the CTL, is a T lymphocyte that kills cancer cells and other infected cells. They promote chosen target cell death with the use of granule and receptor-mediated mechanisms. They have exquisite specificity for antigens that recognize T-cell receptors on target cells and present antigen derived peptide fragments that appear on the surface of the cell to be inserted into the groove of class 1 major histocompatibility molecules [46]. CTLs are attractive as mediators of antitumor immunity that can recirculate throughout the body and seek out antigens, which can be utilized in the treatment of systemic disease. The MHC class 1 complex activates cytolysis through recognition of a single peptide. CTLs employ non-effector mechanisms along with the production of interferon gamma, a cytokine comprising several antitumor properties [47,48]. Cancer cells have a natural homologous adhesion property for tumor targeting. Homotypic binding is the mechanism by which cancer cells adhere to one another, thus allowing tumor growth. Stem cells possess self-renewable capacity with high replicative potential in multilineage differentiation capacity [27]. For therapeutic purposes, embryonic stem cells are generally used due to their advanced totipotency and indefinite lifespan. Tumors secrete vascular endothelial growth factor (VEGF) for the recruitment of mesenchymal stem cells in the formation of supporting stroma for tumors and pericytes aimed for angiogenesis [36]. In general, the membranes obtained from blood cells has innate property of immune escape and ability to transverse endothelium and those from immune cells provides prolonged systemic circulation by their ability to avoid immune clearance. When stem cells become the source for membrane coating, it helps to target cancer and cancer cell sources provides homologous tumor targeting because of its homotypic binding. Table 1 summarizes the general types of source cells and their inherent properties on membrane-coated NPs.

### 2.2. Core Nanoparticles

The nanoparticle used as the core can be either organic or inorganic. Core NPs play an important role in cell-camouflaged NPs, as they are the payload that needs to be delivered to the target site. This core is then shielded by cell membranes isolated from source cells described above.

#### 2.2.1. Organic NPs

Organic NPs are obtained from organic compounds such as lipids and polymers [58]. This type of organic core NP is synthesized either by emulsification process or precipitation method.

##### Poly(Lactic-*co*-glycolic acid) (PLGA) NPs

Poly(lactic-*co*-glycolic acid) (PLGA) NPs are the commonly used polymeric core NPs. This Food and Drug Administration (FDA)-approved polymeric core is biodegradable, biocompatible and non-toxic [59]. These polymeric NPs, when encapsulated with some anticancer drugs or near-infrared (NIR) dye, allow them to be used in image–guided tumor therapy. Luk et al. [21] used PLGA cores for encapsulating the anticancer drug doxorubicin (DOX) and further camouflaged it with RBC membrane via the sonication method. The PLGA cores with highly negative carboxyl groups were shielded by a less negative membrane layer of RBC [21]. In another study, Gao et al. [52] encapsulated perfluorocarbons into PLGA NPs and further camouflaged them with RBC membrane by a co-incubation approach. This RBC-mimicking nanosystem is of smaller size than native RBCs and leaks out from blood vessels and diffuses into solid tumors, thereby increasing oxygenation in tumor regions and favoring cancer treatments that involve reduced hypoxic conditions [52]. In another study by Tian et al. [18], a cancer cell membrane-coated nanocarrier was developed in which the PLGA carrier was co-loaded with hemoglobin and doxorubicin and then further camouflaged with cancer cell membranes through extrusion to overcome hypoxia-induced chemo-resistance and targeted delivery of chemotherapeutic drug [18]. Chen et al. [35] reported a low dose irradiation-guided drug delivery system in which PLGA NPs loaded with the anticancer drug paclitaxel were camouflaged with human cytotoxic T lymphocyte membranes, including the mechanism of coating. The aforementioned biomimetic system enhances the drug targeting ability in gastric cancer [35].

##### Gelatin

Gelatin, a mixture of peptides and proteins, is another type of organic carrier used to coat stem cell vesicles. Bone marrow-derived stem cell membrane-coated gelatin nanogels were developed by Gao [36] and his group, offering efficient tumor targeting delivery of the anticancer drug doxorubicin. Anticancer doxorubicin was loaded into gelatin nanogels and fused with stem cell vesicles via extrusion, and the final fused nanogels mimicked the natural stem cell membrane [36].

##### Liposomes and Human Serum Albumin

Liposomes are also used as core materials for biomimetic systems. They are made by dispersing phospholipids in water and are known for their loading capability and co-delivery of both hydrophilic and hydrophobic drugs [11]. The anticancer drug emtansine was loaded into the pH-responsive liposome and coated with macrophage membrane via extrusion to improve the targeting ability and suppress metastasis in lungs [41]. Another organic core nanoparticle used was human serum albumin (HSA), which is the most abundant protein in human blood plasma. HSA was coated by RBC membranes through extrusion for prolonged circulation. HSA core was encapsulated by the NIR dye Indocyanine green (ICG) and oxygen self-enriched perfluorotributylamine (PFTBA), which can increase singlet oxygen production. This biomimetic system has enhanced phototherapy for cancer treatment [50].

#### 2.2.2. Inorganic NPs

Inorganic NPs are synthetic NPs that are used as cores and further camouflaged with membrane vesicles. Inorganic NPs are known to exhibit electrical, magnetic and optical properties and can be tailored by controlling the shape, size and surface interactions of the NPs [60].

##### Silica

Silica NPs are known to be good carriers due to their highly compatible nature, well-defined mesoporous and hollow structure, and sustained drug release. A study by Li et al. [61] used silica nanorattle as core and encapsulated DOX. Silica nanorattle was bioconjugated with monoclonal antibody, which will allow specific binding for human mesenchymal stem cells. Tumor targeting was initiated by stem cells with tumor tropic properties, and increased intra-tumoral drug distribution and enhanced tumor cell apoptosis was obtained with silica nanorattle encapsulated DOX [61]. He et al. modified one side of the Janus microcapsule with leukocyte membrane for defined tumor targeting, including the mechanism of the coating, and the other side was functionalized with noble metals such as gold nanoparticles for the photothermal killing of cancer cells [31].

##### Magnetic Nanoparticles

Magnetic nanoparticles are those NPs that have magnetization properties under the influence of an external magnetic field and find applications in magnetic resonance imaging (MRI) and alternate magnetic field mediated hyperthermia. Fu et al. [62] used magnetic *O*-carboxymethyl-chitosan (CMC) nanoparticles as cores and camouflaged them with RBC membranes by repeated extrusion [62]. This RBC membrane was anchored with Arg-Gly-Asp, which in turn inhibited tumor growth. The CMC core was co-encapsulated with the hydrophobic–hydrophilic anticancer drugs paclitaxel (PTX) and DOX [62]. In another study, magnetic iron oxide nanoparticles were used as cores, and further coating with RBC membranes via extrusion enhanced the magnetic resonance (MR) intensity and provided prolonged circulation by reducing accelerated blood clearance [16]. Magnetic nanoparticles could be coated with RBC membranes through microfluidic electroporation, in which RBC vesicles and magnetic nanoparticles are merged and flowed through an electroporation zone in a microfluidizer chip. The biomimetic nanoparticles obtained showed longer circulation in blood, increased MR intensity and photothermal property for MR image-guided photothermal therapy against cancer [53].

##### Up-Conversion Nanoparticles

Up-conversion nanoparticles have the ability to convert NIR light to visible light and find applications in fluorescence imaging with unique optical properties such as narrow emission peaks, low toxicity and good photostability [63]. These NPs have remarkable light-penetration depth, less toxicity, negligible background fluorescence, and exceptional photostability [64]. These NPs can be used as core nanoparticles and further coating with macrophage membranes paves their way for use in tumor imaging [56].

The choice of core NPs whether its polymeric/liposomes/inorganic depends on the properties of the cargoes that need to be delivered. Polymeric nanoparticles are used for the sustained delivery of hydrophobic drugs and liposomes are used for rapid drug release of hydrophilic drugs. Inorganic nanoparticles engineered into different shapes and sizes are used to regulate the pharmacokinetic behavior of the nanoparticles. In general, choosing the core NP is very crucial as it decides the drug-releasing property and the pharmacokinetic behavior [38].

**Figure 2:**
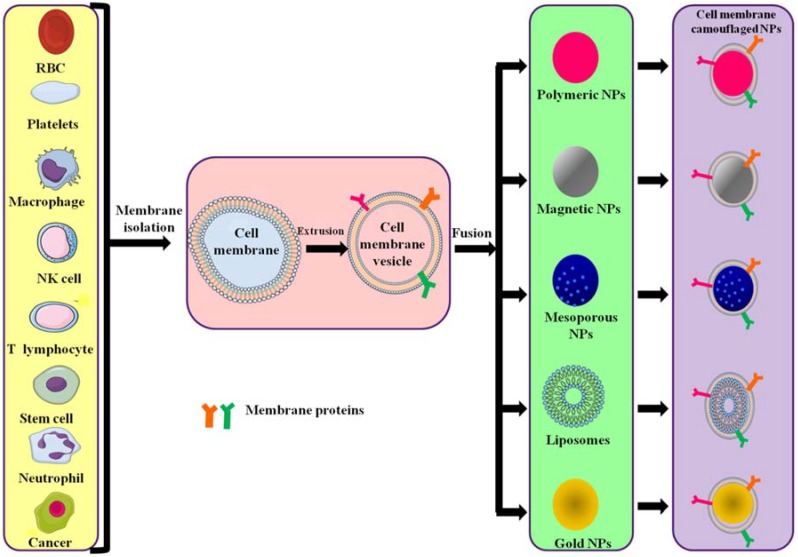
Different source cells and types of polymeric NPs formed by camouflaging different membranes. Cell membranes are isolated from their source cells and extruded to obtain membrane vesicles. The vesicles then fuse with core NPs to form membrane-camouflaged nanoparticles.

The preparation of cell membrane-camouflaged nanoparticles comprises three steps: isolation of membrane vesicles from the source cell, design of the core nanoparticle and fusion of membrane vesicles and core nanoparticle, forming a core–shell structure (Figure 2). All preparation steps are crucial in the design of cell membrane-coated nanoparticles.

*Isolation of membrane lipid bilayer from source cells*: cell membranes/plasma membranes/cytoplasmic membranes are semi-permeable phospholipid bilayer structures with embedded proteins that separate the cell cytoplasm from the outside environment. The isolation of plasma membranes from source cells mainly involves the emptying of intracellular components through a series of steps that includes cell lysis, mechanical disruption and differential ultracentrifugation [65]. Generally, cell membranes are lysed by a hypotonic lysis buffer, freeze–thaw method, mechanical disruption via dounce homogenizer [33], sonication [14,52,66], extrusion [16,18,36] and finally differential centrifugation at very high speeds (approximately 100,000× *g*). The final obtained pellet is resuspended in phosphate buffered saline (PBS) and then lyophilized.

## 3. Formulation Strategy for Cell Membrane-Camouflaged NPs

**Figure 3:**
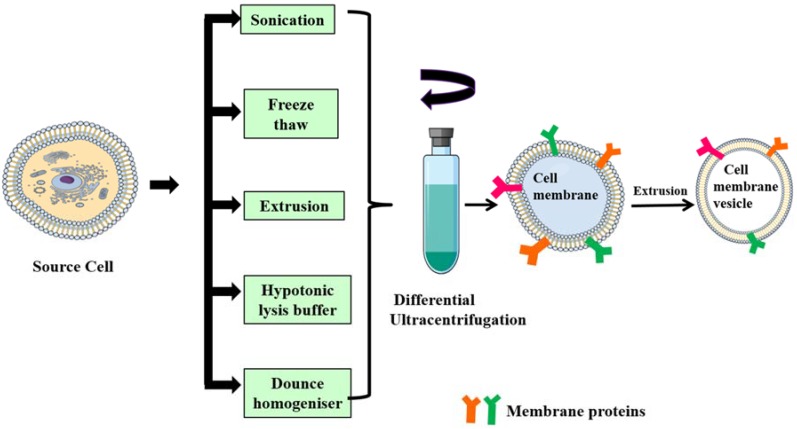
Schematic representation of different methods for isolation of the cell membrane from source cells. Membranes are isolated from source cells through different approaches such as sonication, freeze–thaw, extrusion, hypotonic lysis and dounce homogenizer, and are then subjected to a series of ultra-centrifugations and extruded to obtain membrane vesicles.

*Fusion of core NPs and membrane vesicles*: isolated membrane vesicles are fused with core NPs either by extrusion, sonication or electroporation. In the case of extrusion, both membrane and core NPs are coextruded multiple times through a porous membrane (polycarbonate, polyester) [21,50]. Sonication involves simple co-incubation of membrane vesicles and core NPs under ultrasonic waves, resulting in membrane-coated NPs, but a drawback of this method is that the membrane coating formed might not be uniform and might result in polydisperse particles [32]. Electroporation, on the other hand, induces several pores on the cell membranes due to exposure to strong external electric field. Core NPs diffuse inside cells through these pores. The use of microfluidic chips improves transfection efficiency and membrane vesicles, and core NPs are merged at an electroporation zone in the microfluidic chip [53]. Figure 3 describes the various methods for isolation of cell membrane from their respective source cells, which is then subjected for differential ultracentrifugation. After a series of ultracentrifugation steps, the obtained pellet which is the plasma membrane is extruded to become a vesicle. The obtained vesicles then fused with core NP for obtaining membrane-coated nanoparticles.

## 4. Cellular Membrane Coatings: Different Source Cells and Their Mechanisms

Coating of nanoparticles with cellular membrane preserves the physicochemical properties of nanoparticles and retains the cellular membrane functions of the source cells. They escape immune clearance and promote tumor targeting ability.

### 4.1. Blood Cell Membrane-Camouflaged NPs

**Figure 4:**
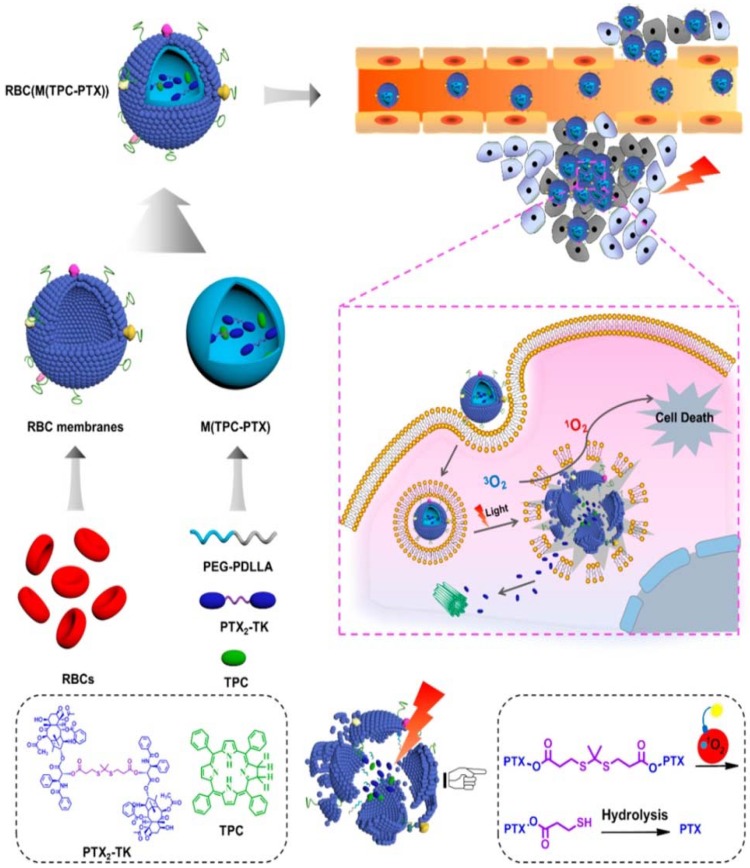
Schematic showing red-blood cell (RBC) membrane-coated dimeric prodrug NPs (RBC(M(TPC-PTX))); light triggered on-demand drug release and combined chemo-photodynamic therapy. The inner components (i) reactive-oxygen species (ROS)-responsive paclitaxel (PTX) dimer with thioketal (TK) as the linker and 5,10,15,20-tetraphenylchlorin (TPC) loaded in methoxypoly(ethylene glycol)-block-poly(d,l-lactide) (PEG-*b*-PDLLA) NPs for drug retention and light-triggered amplified drug release; (ii) RBC membrane-based outer shell for long blood circulation. Reproduced with permission from Ref [67]. Copyright © 2018 American Chemical Society.

Blood cells generally comprise red blood cells (erythrocytes), white blood cells (leucocytes) and platelets. There are many studies on membranes derived from these different types of blood cells [12,35,37,56]. Different source cells used to isolate membranes are explained in Section 2.1. RBC membrane-coated nanoparticles were the first reported membrane-camouflaged nanoparticles. RBC coated nanoparticles translocate the protein makeup on the RBC surface and reduce accelerated blood clearance. In the first report of RBC membrane-coated NPs, RBC membranes were coated onto negatively charged PLGA nanoparticles. In this study, the RBC membrane was derived through hypotonic treatment, and the inclusion of membrane over NP occurred through extrusion [30]. Ding et al. [13] developed RBC membrane-camouflaged up-conversion nanoparticles for photodynamic therapy. They used folic acid for tumor targeting, triphenyl phosphonium for mitochondrial targeting and encapsulated photosensitizer merocyanine 540 (MC 540). MC 540 generates singlet oxygen upon 980 nm laser irradiation based on the light-transducing ability of up-conversion nanoparticles, thus enhancing the therapeutic efficacy [13]. Recently, Liu et al. [17] reported that RBC biomimetic membranes coated near-infrared luminescent nanophosphors with persistent luminescence and were excellent biocarriers for the delivery of doxorubicin. They used triple-doped zinc gallogermanate nanostructures for super long-persistent luminescence and coated RBC membranes [17]. RBC membrane-camouflaged nanoparticles are also used in synergistic photodynamic and chemotherapy [49,67]. Dimeric prodrug nanoparticles can be activated by light for efficient drug release for effective photodynamics and chemotherapy. The dimeric prodrug is composed of a paclitaxel dimer and tetraphenylchlorin for drug retention and is activated by light for enhanced drug release (Figure 4) [67]. RBCs contain oxyhemoglobin, which could be used to enhance the efficiency of photodynamic therapy. Wan et al. [49] has reported this use of oxyhemoglobin to enhance photodynamic therapy. In this study, RBC membranes were camouflaged, combined with the CO_2_ gas-generating agent ammonium bicarbonate and loaded with ICG and DOX for effective phototherapy and chemotherapy [49]. Melanin, usually present in skin, is a natural biopolymer and acts as an effective photothermal agent with high photothermal conversion efficiency and used in multimodality imaging [6,68,69]. Functionalizing melanin nanoparticles with RBC membranes makes this biomimetic system suitable for in vivo cancer photothermal therapy, and melanin as the inner core acts as a photoacoustic (PAI) contrast agent [51].

**Figure 5:**
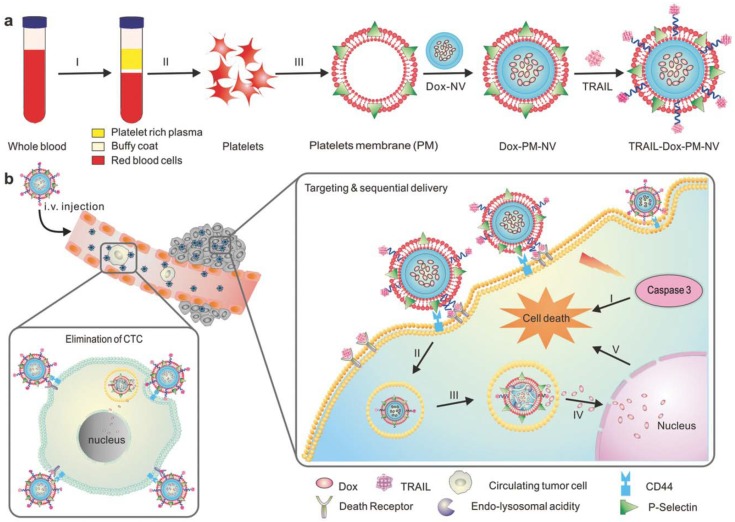
Schematic showing mechanism of drug loaded platelet membrane coated nanovehicle for targeted and sequential drug delivery. (**a**) Isolation and extraction of platelet membranes, coating it over core doxorubicin (DOX) nanovehicle (DOX_NV) and TRAIL conjugated platelet membrane; (**b**) TRAIL-DOX nanovehicle (NV) capture the circulating tumor cells by specific affinity of P-selectin and CD44 receptors in cancer and triggers the sequential release of TRAIL-DOX NV. (I) Interaction of TRAIL and death receptors initiates apoptosis signals and (II) internalization of TRAIL-DOX NV (III) TRAIL-DOX NV dissociation by acidity of lyso-endosome (IV) release of DOX in nucleus (V) DOX induced intrinsic apoptosis. Reproduced with permission from reference [70]. Copyrights © 2015 John Wiley and Sons.

Another important component of blood that is essential for maintenance of homeostasis is platelets. In the case of platelets, the membranes obtained carry the surface moieties, antigens and proteins present on the native platelets. Platelet membrane-camouflaged nanoparticles have various biomedical applications, including drug delivery [20], cancer treatment [40,70] and treatment of immune thrombocytopenia [71]. Platelets contain the protein P-selectin that is overexpressed on its membrane and specifically binds to CD 44, which is upregulated in cancer cells [72]. This makes platelet membranes suitable for anticancer therapy, as this membrane coating helps in tumor targeting and subsequent delivery of the payload, thus preventing the sudden burst release of payload. Recently, Xu et al. [40] developed a biomimetic system with PLGA nanoparticles loaded with the photodynamic agent verteporfin as the core and further camouflaging with platelet membrane. The article claims that the photodynamic nanoparticles have active targeting capacity, bring the tumor cells within the ROS reach and help in effective tumor therapy without causing skin damage [40]. In another study by Jing et al. [34], arginyl-glycyl-aspartic acid (RGD) peptide modified platelet membranes were used to camouflage melanin nanoparticles and doxorubicin (DOX). The melanin nanoparticle along with DOX had chemo-photothermal therapy. Platelet membranes modified with RGD peptides have immune evading capabilities due to platelet membranes and the targeting ability of RGD peptide to αvβ3 integrin, thus paving the way to target tumor vasculature [34]. Hu et al. [70] developed platelet-membrane coated nanovesicles loaded with two anti-cancer drugs DOX and TNF related apoptosis inducing ligand (TRAIL). TRAIL binds to death receptors on a cell surface and induces apoptosis whereas DOX triggers intrinsic apoptotic signaling by intercalating with DNA. P-selectin on platelet membranes binds to CD44 receptors on cancer cells and thus captures circulating tumor cells. Thus, the platelet membrane-camouflaged TRAIL-DOX nanovesicles aids in targeted and sequential drug delivery causing extrinsic and intrinsic apoptosis (Figure 5) [70].

Another class of blood cell membrane is from leukocytes, which aid the transport of chemotherapeutics across the endothelium by preferential binding to inflamed endothelium, thus escaping the lysosomal pathway [73]. The first report on leukocyte membrane-camouflaged NPs was by Parodi et al. [73]. In this study, they isolated leukocyte membranes from freshly isolated leukocytes and camouflaged naked, porous silicon nanoparticles. This synthesized biofunctionalized nanoparticle avoided opsonization and delayed uptake by the mononuclear phagocyte system [73]. In his article, He et al. [31,32] presented leukocyte membrane modified Janus microcapsules in which one part of the Janus microcapsule is coated with gold and the other part is coated with THP-1 human monocyte leukemia cell membrane. These bio-functionalized Janus capsules could discriminate different cancer cells from non-cancerous cells and used as photoactive cancer cell detector to kill cells based on the photothermal property of gold [31,32].

### 4.2. Immune Cell-Camouflaged NPs

**Figure 6:**
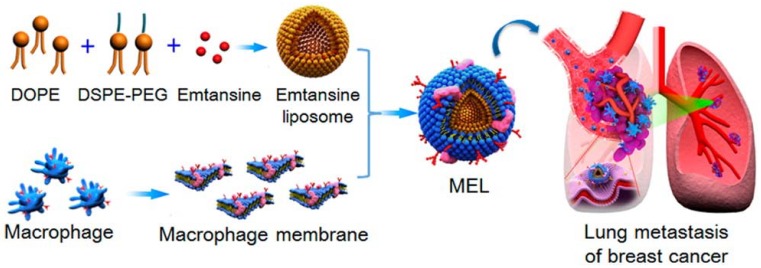
Scheme of macrophage-membrane-coated emtansine liposome with specific metastasis targeting for suppressing lung metastasis of breast cancer. Reproduced with permission from reference [41]. Copyright ©2016 American Chemical Society.

Immune cells such as macrophages, neutrophils, cytotoxic T cells, and NK cells are used as source cells to isolate their membrane and functionalize synthetic nanoparticles, paving the way for a wide range of applications in tumor therapy. The camouflaged macrophage membrane contains the associated membrane proteins of natural macrophages, thus making it ideal for tumor targeting and imaging [56]. Macrophages contain α4 integrins, which allow them to actively bind to VCAM-1 (vascular adhesion molecule) on cancer cells. Macrophage membranes are derived by emptying the intracellular contents through the combined disruption methods of hypotonic lysis, mechanical disruption and a series of differential centrifugation steps. Further coating of this membrane on synthetic nanoparticles occurs through extrusion. The applications of these membrane-camouflaged nanoparticles include photothermal tumor therapy, with the core particle being a photothermal agent whose optical absorption lies in the NIR region, and other factors such as biocompatibility, decreased opsonization, prolonged circulation and enhanced tumor accumulation [55]. Xuan et al. [55] reported macrophage membrane-camouflaged gold nanoseeds for photothermal cancer therapy. These gold nanoseeds were formed through a seed-mediated growth method on the surface of silica nanoparticles. They carried NIR dye cyanine 7 and macrophage membranes obtained from freshly harvested macrophages [55]. In another study, macrophage membranes were decorated on liposomes in order to target and suppress lung metastasis of breast cancer. Macrophage membranes were derived from RAW 264.7 cells with high expressions of α4 and β1 integrins. A pH-sensitive liposome was loaded with the anticancer drug emtansine and further coated with isolated macrophage membranes (Figure 6) [41]. In a recent work by Zhang et al. [25] and his group, a macrophage membrane-coated nanosystem was developed in which the core nanoparticle was a pH-sensitive polymer functionalized with a cationic 2-aminoethyldiisopropyl group, which helps to tune its buffer capacity to the extracellular pH of the tumor environment. The polymer was conjugated with insulin-like growth factor 1 (IGF1R) targeting peptide. Further co-extrusion with freshly harvested macrophage membrane results in a membrane-camouflaged nanoparticle that has a proton sponge effect and shows step-by-step drug release in response to different pH in the tumor microenvironment, tumor homing ability and increased tumor-targeted chemotherapy [25].

**Figure 7:**
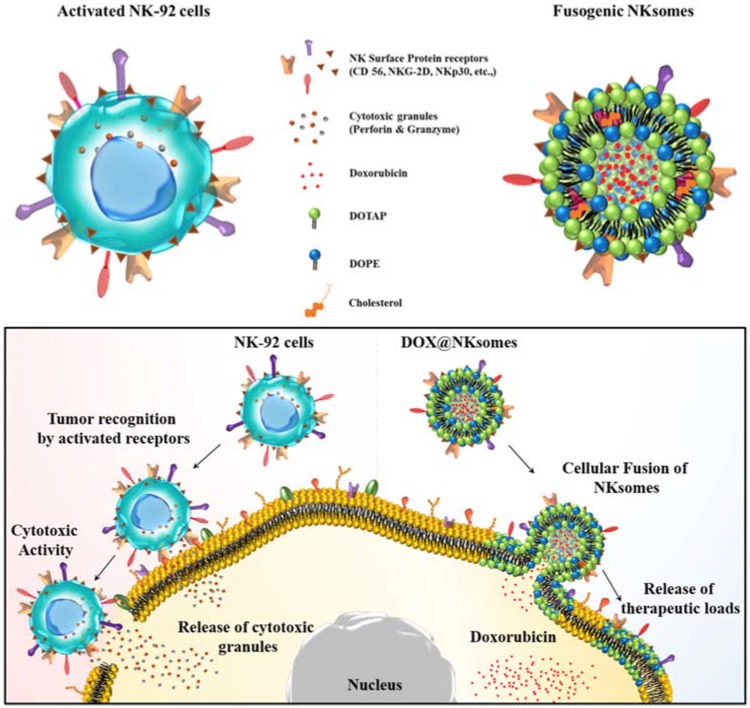
Schematic illustration of NK cells and NK cell membrane-derived fusogenic liposomes (NKsomes). Fusogenic liposomes recognize tumor cells and help release the therapeutic loads. Reproduced with permission from Ref [26]. Copyright © 2018 Elsevier Ltd. All rights reserved.

The antitumor response compromises with increased numbers of circulating neutrophils, which in turn has a negative impact on the cytotoxic activity of NK cells and lymphocytes [74]. Neutrophil camouflaged nanoparticles are used to target circulating tumor cells (CTCs) in circulation. Further incorporating nanoparticles that have second-generation proteasome inhibitor allows therapeutic applications by preventing de novo metastasis and inhibition of already formed metastasis [33]. NK cell membrane-camouflaged cationic liposome was loaded with DOX and exhibited tumor-homing potential and targeted tumor therapy. This NK cell membrane along with its membrane proteins were isolated and extruded with liposome and loaded with DOX to form DOX-loaded NKsomes (Figure 7). This activated NKsome has surface receptors such as NKG2-D, NKp30 and NKp44 for cytolytic functions [26].

The properties of CTL are explained in the above section. Cytotoxic T cell membrane-camouflaged nanoparticles could be used as tumor-targeting nanoparticles. Zhang et al. [35] and his coworkers isolated human cytotoxic T lymphocyte membranes and coated over PLGA nanoparticles encapsulated with paclitaxel. Upon exposure to local low dose irradiation, this biomimetic system is localized at the tumor site and releases chemotherapy drugs [35].

### 4.3. Cancer Cell Membrane-Camouflaged Nanoparticles

**Figure 8:**
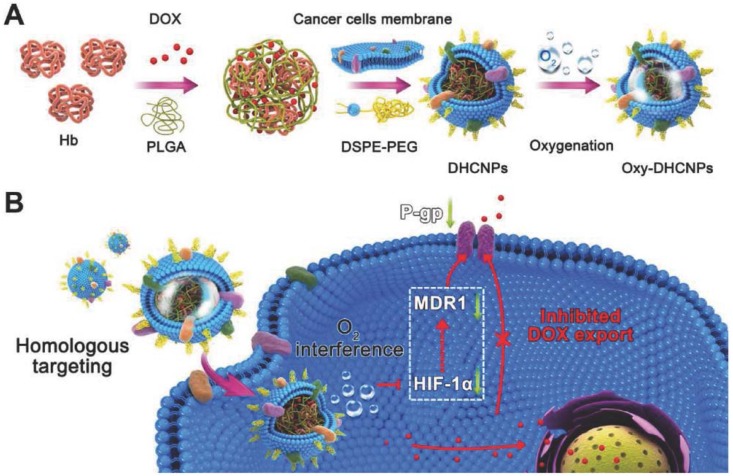
Cancer cell membrane-camouflaged poly(lactic-*co*-glycolic acid) (PLGA) NPs were loaded with DOX and hemoglobin for homotypic targeting and O_2_ interference. (**A**) Synthesis of hemoglobin (Hb) and DOX-loaded PLGA NPs coated with cancer cell membranes and DSPE-PEG (1,2-distearoyl-*sn*-glycero-3-phosphoethanolamine-*N*-maleimide), provides homo targeting; (**B**) Cellular functions of Dox/Hb loaded PLGA-cancer cell membrane nanoparticles (DHCNPs), including homologous targeting, downregulation of predictive markers (HIF-1α, MDR1, and P-gp), and inhibited DOX export. Reproduced with permission from Ref [18]. Copyright © 2016, John Wiley and Sons.

Functionalizing nanoparticles with cancer cell membranes aid homotypic targeting, which in turn facilitates internalization by source cells because of its self-recognition property [19]. Homotypic cell membranes increase chances for particle-to-cell adhesion and have potential capability in targeting different sites that are vulnerable to cancer metastasis. Cancer cell membrane camouflaged NPs have similar cell adhesion molecules as their source cells. Because of this, nanoparticles camouflaged with cancer cell membranes can be utilized for a wide range of applications for anticancer vaccination, drug delivery, and image-guided photothermal therapy to increase the therapeutic effect by targeted oxygen interference for chemoresistance [18,19,27,57]. Cancer cell membrane-camouflaged NPs allow tumor-associated membrane bound antigens with immunological adjuvants to effectively deliver to antigen-presenting cells by stimulating anticancer immune responses. The ability of cancer cell membrane-coated NPs to initiate homologous targeting with inherent self-adhesive properties allows delivery of different anticancer drugs to the tumor site. Fang et al. [57] reported successful functionalization of polymeric NPs with cancer cell membrane and delivery of antigens with immunological adjuvants for enhancing tumor-specific immune response in anticancer vaccination [57]. Cancer cell membrane-coated NPs exhibited increased tumor accumulation through specific homotypic targeting and passive targeting by enhanced permeability and retention (EPR) effect. Polymeric PLGA NPs loaded with ICG become the core, and further coating with the cancer cell membrane provides dual modal (fluorescence/photoacoustic) image guided photothermal therapy. The cancer cell membranes are isolated from source cells through a combination of hypotonic lysing of cells, mechanical disruption by sonication, freeze–thaw, dounce homogenizer and differential centrifugation [19,27,57]. The obtained pellet is then coated over the core nanoparticle by co-extruding the membrane with the nanoparticle. Magnetic core NPs are used for MRI applications, Zhu et al. [19] reported that magnetic iron oxide nanoparticles coated with different types of cancer cell membranes achieved specific self-recognition to the source cells in competition with another heterologous tumor. In this study, doxorubicin-hydrochloride (DOX-HCl) was attached to negatively charged magnetic iron oxide nanoparticles through electrostatic interaction, which aided MR image-guided chemotherapy. In another study, cancer cell membranes camouflaged over polymeric PLGA cores with hemoglobin and anticancer drug DOX possessed selective targeted delivery of chemotherapeutic drug for enhanced chemotherapy and oxygen to homologous tumors, thus breaking hypoxia-induced chemo-resistance (Figure 8) [18]. PTX-loaded polymeric poly(caprolactone) PCL NPs coated with 4T1 breast cancer cell membranes showed enhanced targeting efficiency and delivery of the anticancer drug PTX for chemotherapy of metastatic cancer [37].

Zhang et al. [75] and his coworkers developed biomimetic magnetosomes as artificial antigen presenting cells (APCs) that provide high activity and efficiency for cytotoxic T-cell expansion and reinfusion to tumor tissues through MR image guidance and magnetic control. In this study, leukocyte membranes were pre-engineered with azide via intrinsic biosynthesis, underwent phospholipid incorporation and were electrostatically coated onto magnetic nanoparticles. T-cell stimulatory signals (peptide-loaded major histocompatibility complex class-I and anti-CD28) were modified with dibenzocyclooctyne (DBCO) and decorated onto magnetosomes through the copper-free click chemistry method. Artificial antigen presenting cells (APC-)CTLs were delivered by MR image guidance and magnetic control, providing T cell-based anticancer immunotherapy [75].

### 4.4. Stem Cell Membrane-Camouflaged NPs

**Figure 9:**
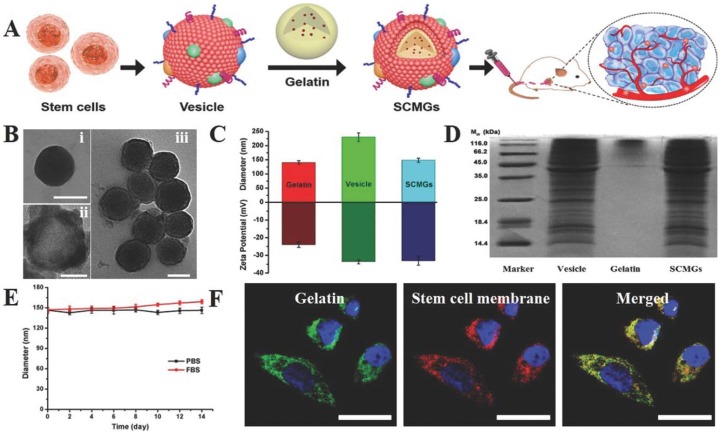
Stem cell membrane-camouflaged gelatin (SCMGs) nanogels loaded with DOX for tumor-targeted delivery of anticancer drugs in vivo. (**A**) Isolation of membranes from stem cells and encapsulation of DOX-loaded gelatin nanogels; (**B**) characterization of stem cell membrane-camouflaged nanoparticles. Transmission electron microscope (TEM) images of (i) bare gelatin nanogel, (ii) stem cell membrane vesicle and (iii) stem cell membrane-coated nanoparticle. Scale bar = 100 nm; (**C**) hydrodynamic particle size and surface zeta potential of bare gelatin nanogels, stem cell membrane vesicles and stem cell membrane-coated nanoparticles; (**D**) sodium dodecyl sulfate–polyacrylamide gel electrophoresis (SDS-PAGE) protein analysis of stem cell membrane vesicles; (**E**) stability of SCMGs in PBS and FBS; (**F**) colocalization of gelatin nanogels (fluorescein isothiocyanate, FITC channel) and stem cell membranes (1,1′-dioctadecyl-3,3,3′,3′-tetramethylindo-dicarbocyanine, DiD channel) shown by confocal laser scanning microscopy, CLSM images. Scale bar = 20 μm. Reproduced with permission from Ref [36]. Copyright © 2016, John Wiley and Sons.

Stem cell membrane-camouflaged nanoparticles have an intrinsic tumor tropic property that makes them suitable for tumor therapy [36]. Mesenchymal stem cell membranes derived from bone marrow were coated on gelatin nanogels loaded with the anticancer drug DOX for efficient tumor-targeted drug delivery. These gelatin nanogels consisted of a unilamellar membrane functionalized with tumor-targeted antigen associated with stem cells. Mesenchymal stem cell mimicking tumor-targeting capability was showcased in vitro and enhanced tumor accumulation in vivo (Figure 9). The tumor tropic property is well preserved and reticuloendothelial system (RES) clearance is decreased due to this functionalization with stem cell membranes [36].

### 4.5. Hybrid Cell Membrane-Camouflaged NPs

**Figure 10:**
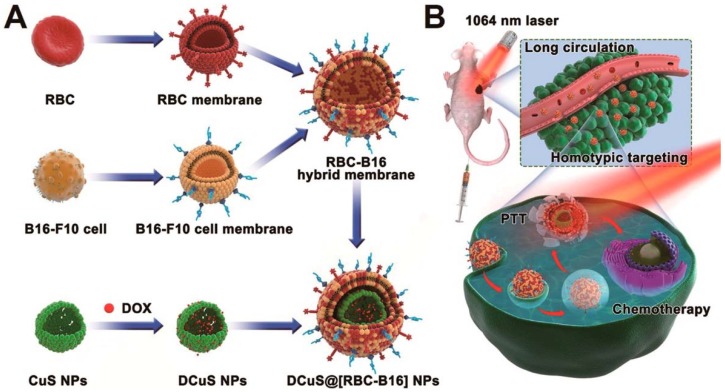
Schematic showing the fusion of RBC and cancer cell membranes and camouflaging Doxorubicin loaded hollow copper sulfide nanoparticles and its synergistic near infrared (NIR) laser-triggered chemo/photothermal therapy. Reproduced with permission from reference [76]. Copyright © 2018, American Chemical Society.

In hybrid cell membrane-camouflaged NPs, multiple functionalities can be incorporated within a single platform by the fusion of cell membranes from different cell sources [14,76]. This strategy of synthesizing multi-membrane nanoparticles increases the functionality for use in specific applications. Dehaini et al. [14] reported the first RBC-platelet hybrid membrane-coated PLGA nanoparticle for enhanced functionalization. RBC membranes provided prolonged circulation and platelets provided tumor-targeting ability. Fusion of dual membranes was carried out by doping the platelet membranes with two different dyes that constituted Förster resonance energy transfer (FRET). Further fusing of RBC membranes onto this decreased the fluorescence intensity between the two dyes, indicating that RBC membranes fused in between platelet membranes. RBC membranes labeled with fluorescent dye and platelet membranes with the FRET pair of dyes showed localization after fusing. Wang et al. [76] and his group reported a hybrid membrane coating of RBC and cancer cell membranes for targeted chemo-photothermal therapy of melanoma. In this, RBC-cancer hybrid membranes were coated onto DOX-loaded hollow copper sulfide nanoparticles. Cancer cell membranes were doped with a pair of FRET dyes, and addition of RBC onto this decreases the fluorescence intensity of the FRET dyes. In the resultant RBC-cancer cell hybrid membrane-coated nanoparticle, RBC membranes helped with prolonged circulation, cancer cell membranes provided homotypic targeting, and copper sulfide with DOX loading allowed for combined chemo/photothermal therapy (Figure 10) [76].

From the above section, the different types of source cells with their own unique characteristics is explained. Cell membrane camouflaged NPs have emerged as a potential strategy in the treatment of cancer therapy as variety of membrane camouflaged nanoparticles could be developed based on the type of source cells used. The most promising factor is that it is recognized as same as that of their respective source cells which provides them advantages like prolonged circulation and immune evasion capability which could be used in cancer theragnostics.

## 5. Applications of Cell Membrane-Camouflaged Nanoparticles

Cell membrane-coated nanoparticles have found a wide range of applications because of their biocompatibility, prolonged blood circulation, and tumor-targeting ability based on their coating membranes. Based on the type of surface markers, membrane coatings pave the way for different applications. In this section, the applications of membrane-coated NPs in tumor therapy are briefly discussed.

### 5.1. Drug Delivery

Various types of membrane coating were used for targeted drug delivery. RBC-NPs were used to deliver DOX for the treatment of solid tumors [21]. RBC membrane coating over the hydrophilic–hydrophobic anticancer drugs DOX and PTX was used for combined chemotherapy. They co-encapsulated both hydrophobic and hydrophilic chemotherapeutic drugs into magnetic *O*-carboxymethyl chitosan particles that hide in the bloodstream, are magnetically activated and accumulate in tumor cells, releasing drugs into the cytoplasm. The erythrocyte membrane vesicles were coated onto the NPs through a series of extrusions [62]. RBC membranes modified by pre-inserting streptavidin and incorporating a biotinylated form of ^D^CDX peptide possessed the capability to cross the blood–brain barrier, which can further be used to deliver DOX against brain glioma [22]. RBC membrane coatings over up-conversion nanoparticles (UCNPs) used as photodynamic therapy (PDT) agents enabled targeted drug delivery and phototherapy [13]. Monoclonal antibodies are coated with RBC membranes, and different types of antibodies are intracellularly delivered [77]. pH-sensitive liposomes coated with macrophage membranes successfully delivered the anticancer drug emtansine against lung metastasis of breast cancer [41]. Platelet-coated NPs with docetaxel and vancomycin are used for disease-targeted therapy [43].

### 5.2. Near Infrared (NIR) Phototherapy

In NIR phototherapy, selective and localized therapeutic effects can be accomplished with the help of laser irradiation. NIR phototherapy consists of two treatment modalities: photothermal therapy and photodynamic therapy. Photothermal therapy uses near NIR laser photo absorbers for thermal ablation of cancer cells through laser irradiation. The latter uses a photosensitizer that is excited at a specific band light and generates singlet oxygen, which creates local hyperthermia to kill cancer cells. Different membrane-coated nanoparticles enhance phototherapy.

#### 5.2.1. Photothermal Therapy

**Figure 11:**
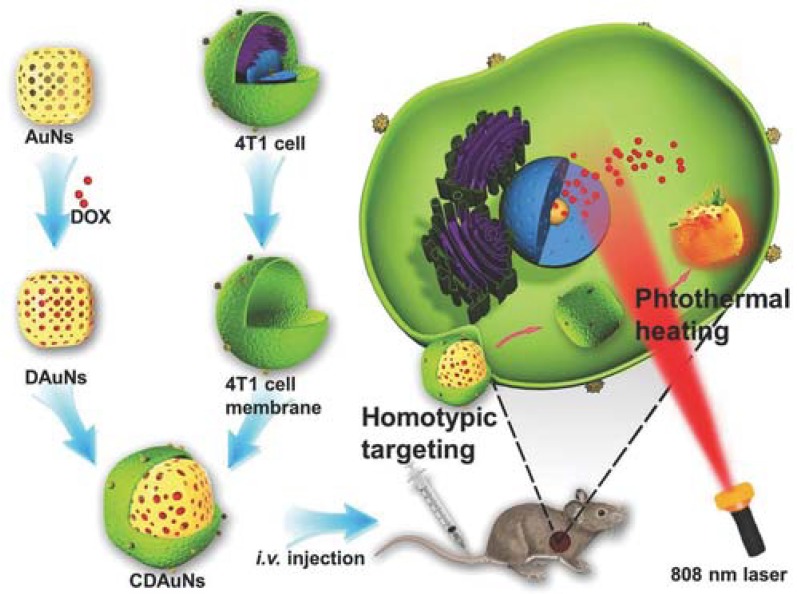
Schematic showing 4T1 cancer cell membrane coated over gold nanocage loaded with doxorubicin for NIR triggered release of doxorubicin and homologous targeted therapy of breast cancer. Reproduced with permission from reference [37]. Copyright © 2016, John Wiley and sons.

In one study, magnetic NPs were forced to enter RBC vesicles through the electroporation method, and the resulting NPs helped in MR image-guided photothermal therapy [53]. Cancer cell membrane coating on PLGA NPs with ICG through extrusion demonstrated specific homologous targeting towards cancer with excellent fluorescence and photoacoustic imaging-guided photothermal therapy. Briefly, the core NPs were the ICG-loaded PLGA polymeric core, and the cancer cell membrane vesicles were fused on the surface by extrusion. These biomimetic NPs showed excellent mono dispersity, photothermal property, fluorescence/photoacoustic dual-modal imaging properties and homologous tumor targeting ability [27]. Gold nanoshells coated with macrophage membranes (through repeated extrusion) become a photothermal conversion agent for effective photothermal therapy in cancer [55]. Janus capsules modified with gold nanoparticles in one part and leukocyte membrane on the other part were also shown to have specific targeting towards cancer cells and increased photothermal therapy (PTT) [32]. Jing et al. [34] and his group reported a study in which they encapsulated DOX and melanin NPs with RGD peptide-modified platelet membranes for combined chemo-photothermal therapy. Melanin NPs in this platelet-camouflaged nanovesicle system facilitated image guided photothermal therapy and DOX for chemotherapy. Modification with RGD peptide increased the tumor vasculature targeting, as RGD-modified platelet membranes can target αvβ3 integrin present in tumor vasculature [34]. Chen and his coworkers camouflaged cancer cell membranes over PLGA NP loaded with ICG for image-guided photothermal therapy. Cancer cell membrane camouflaging facilitated homotypic targeting, whereas ICG acted as a photothermal agent with fluorescence and photoacoustic properties [27]. In another study, a hybrid membrane was used in which cancer cell membranes and RBC membranes were fused and coated over a copper sulfide nanoparticle loaded with DOX. The key reason for using two types of membranes is that RBC membranes provide longer circulation and cancer cell membranes provide homotypic targeting. This hybrid membrane-camouflaged nanosystem provides combined chemo-photothermal therapy, as copper sulfide has a photothermal conversion property [76]. Zhang et al. [42] reported cracked cancer cell membranes coated over DOX-ICG NPs for combined chemo-photothermal therapy. DOX-ICG NPs were formed by the solvent exchange method and a cracked cancer cell membrane was coated over it. DOX provides a chemotherapeutic effect and ICG acts as a photothermal agent. Further cancer membrane coating facilitates homotypic targeting [42]. Sun et al. [37] developed a nanosystem in which anti-cancer DOX was loaded into gold nanocage and camouflaged with 4T1 breast cancer cell membrane for homologous targeting of breast cancer and NIR laser-triggered photothermal therapy (Figure 11). Coating this nanosystem with cancer cell membrane aids for homologous targeting of breast cancer. DOX which was used for chemotherapy was loaded into the gold nanocages and, upon irradiation, gold nanocages are heated up resulting in hyperthermia-triggered drug release [37].

#### 5.2.2. Photodynamic Therapy

RBC membrane coating onto ICG-human serum albumin (HSA) NPs via extrusion provides prolonged circulation time and increased singlet oxygen generation for photodynamic therapy. In this way, the core HSA NPs can be synthesized by encapsulating ICG and perfluorotributylamine (PFTBA) and can be coated with RBC membrane vesicles through extrusion [50]. In another study on cancer cell membrane coating, phosphorescence image-guided photodynamic therapy was carried out. Herein, a platinum (II) porphyrinic nanoscale metal-organic framework (NMOF) with zirconium (Zr_6_ cluster) in which the porphyrin-NMOF with high photosensitizer loading had O_2_ sensing and phosphorescence-guided PDT [17]. Ding et al. [13] developed RBC membrane-camouflaged up-conversion nanoparticles for photodynamic therapy. They used folic acid and triphenylphosphonium as targeting moieties and encapsulated photosensitizer MC 540 for singlet oxygen generation upon 980 nm laser irradiation based on the light-transducing ability of up-conversion nanoparticles, thus enhancing the therapeutic efficacy [13].

Various applications of cell membrane-coated NPs based on their source cells are explained in Table 2. Cell membrane-camouflaged NPs increase the stability of synthetic materials and enable it to minimize immune clearance. The major advantage of such NPs is its affinity to target cancer cells, thus paving way for targeted drug delivery and phototherapy. Cell membrane-coated NPs can be used as contrast agents in imaging and vaccine which could also be used in cancer treatment [17,78].

## 6. Conclusions and Future Perspectives

Nanoparticles coated with different types of cell membranes could be employed for a variety of biological applications, including drug delivery, phototherapy, photodynamic therapy, imaging applications, and anticancer therapy. Important challenges for membrane-camouflaged nanoparticles are the large-scale production of these NPs without batch-to-batch variation and the scarcity of source cells, including stem cells, within the body. The fusion procedures used must be scalable and optimized for enhanced efficiency. The denaturation of membrane proteins should be eliminated to prevent potential immune responses against endogenous antigens. However, we believe that these issues will be addressed in the near future and that cell membrane-coated nanomedicine will have a great impact on cancer theragnostics.

Membrane coatings help to functionalize nanoparticles by translocating membrane proteins from source cells into the membrane coatings, thus marking NPs as “self”, which in turn contributes to the prolonged circulation of NPs in blood and efficient evasion from immune clearance. Nanoparticles coated with a particular cell membrane will provide homologous targeting and enhanced tumor accumulation. Membrane-coated nanoparticles indeed mimic source cells. Membrane-coated NPs improve the therapeutic efficacy of drugs and other therapeutic cargos via specific delivery and enhanced accumulation of NPs in tumors. Further modification of isolated cell membranes, such as double membrane coating and incorporation of novel therapeutics, endows a new strategy in biomimetic platforms.

## Figures and Tables

**Table 1:** Different source cells and their membrane-coating properties.

Source Cell	Properties in Membrane Coated NPs	Reference
Blood cells	Immune evasion ability	[12,13,16,30,31,32,34,43,49,50,51,52,53,54]
	Ability to transverse endothelium	
Immune cells	Reduce opsonization and prolonged circulation	[25,26,35,41,55,56]
Stem cells	Good cancer targeting property	[36]
Cancer cells	Homotypic targeting	[18,19,27,37,57]

**Table 2:** Application of various membrane-coated nanoparticles.

Type of NPs	Membrane Derivation	Core Particle	Application	References
RBC/erythrocyte-coated NPs	Hypotonic treatment followed by extrusion Sonication approach Hypotonic treatment and sonication Microfluidic electroporation Sonication and extrusion	HSA NPs with ICG and PFTBA Magnetic NPs PLGA NPs PLGA NPs coloaded with DOX Magnetic *O*-carboxymethyl-chitosan nanoparticles coloaded with PTX and DOX PLGA core with PFC DSPE-PEG functionalized UCNPs Oxyhemoglobin and ammonium bicarbonate coloaded with ICG and DOX	Enhanced phototherapy Toxin nanosponge Drug delivery to treat solid tumors Combinational chemotherapy Enhanced radiotherapy Enhanced tumor imaging Combined photothermal/photodynamic chemotherapy	[12,21,49,50,52,54,62]
Platelet coated NPs	Repeated freeze-thaw process followed by sonication Sonication	PLGA NPs with Docetaxel and Vancomycin Si NPs, TRAIL conjugation TRAIL-DOX NPs PLGA NPs PLGA NPs with verteporfin Melanin NPs with DOX	Disease-targeted delivery Photodynamic therapy Phototherapy	[20,34,40,43,70,71]
Leucocyte coated NPs	Coincubation	Janus particles Nanoporous Silicon NPs	Photothermal cancer treatment Drug delivery	[31,32,73]
Neutrophil coated NPs	Percoll gradient separation followed by emulsion/solvent evaporation	PLGA NPs loaded with carfilzomib	Targeting CTCs in circulation and premetastatic niche	[33]
NK cell membrane coated NPs	Extrusion	Fusogenic liposomes with DOX	Targeted drug delivery	[26]
Cytotoxic T lymphocyte membrane coated NPs	Hypotonic lysis and extrusion	PLGA with PTX	Enhanced tumor accumulation and drug delivery	[35]
Macrophage membrane coated NPs	Hypotonic lysis and extrusion	UCNPs Au nanoshells coated mesoporous silica NPs Liposomes with emtansine Targeting polymer conjugated with insulin such as growth factor	Effective cancer imaging Enhanced cancer photothermal therapy Specific metastasis targeting Tumor targeted chemotherapy	[25,41,55,56]
Stem cell coated NPs	Hypotonic lysis and extrusion	Gelatin nanogels	Efficient tumor targeting and drug delivery	[36]
Cancer cell membrane-coated NPs	Extrusion	PLGA core with DOX and Hb PLGA core with ICG PLGA with adjuvants Fe_3_O_4_ and DOX Pt(II) porphyrin nanoscale metal-organic framework with Zirconium cluster Polymeric NP with paclitaxel	Oxygen interfered chemotherapy Photothermal therapy, Photoacoustic imaging Phosphorescence image-guided photodynamic therapy Anticancer vaccination and drug delivery Magnetic Resonance Imaging Tumor targeted chemotherapy	[18,24,27,37,57]
Hybrid cell membrane-coated NPs: RBC-cancer cell membrane RBC-platelet membrane	Sonication	Hollow mesoporous copper sulfide with DOX PLGA core	Combined chemo-photothermal therapy Enhanced nanoparticle functionalization	[14,76]
